# The utility of surgical lung biopsy in cancer patients with acute respiratory distress syndrome

**DOI:** 10.1186/1749-8090-8-128

**Published:** 2013-05-16

**Authors:** Chih-Hao Chang, Kuo-Chin Kao, Han-Chung Hu, Chen-Yiu Hung, Li-Fu Li, Ching-Yang Wu, Chih-Wei Wang, Jui-Ying Fu, Chung-Chi Huang, Ning-Hung Chen, Cheng-Ta Yang, Ying-Huang Tsai

**Affiliations:** 1 Department of Thoracic Medicine, Chang Gung Memorial Hospital, Chang Gung University College of Medicine, Taoyuan, Taiwan; 2 Department of Respiratory Therapy, Chang Gung Memorial Hospital, Chang Gung University College of Medicine, Taoyuan, Taiwan; 3 Department of Respiratory Therapy, Chang-Gung University College of Medicine, Taoyuan, Taiwan; 4 Department of Thoracic Surgery, Chang Gung Memorial Hospital, Chang Gung University College of Medicine, 5 Fu-Hsin Street, Taoyuan, Gweishan Zip 333, Taiwan; 5 Department of Pathology, Chang Gung Memorial Hospital, Chang Gung University College of Medicine, Taoyuan, Taiwan

**Keywords:** Surgical lung biopsy, Cancer, Acute respiratory distress syndrome, Outcomes

## Abstract

**Background:**

This retrospective study evaluated the utility and safety of surgical lung biopsy (SLB) in cancer patients with acute respiratory distress syndrome (ARDS).

**Methods:**

All cases of critically ill patients with cancer and diagnosed with ARDS who underwent SLB in a tertiary care hospital from January 2002 to July 2009 were reviewed. Clinical data including patient baseline characteristics, surgical complications, pathological findings, treatment alterations, and survival outcomes were retrospectively collected and analyzed.

**Results:**

A total of 16 critically ill patients with cancer diagnosed with ARDS who underwent SLB were enrolled. The meantime from ARDS onset to SLB was 3.0 ± 1.5 days. All SLB specimens offered a pathological diagnosis, and specific diagnoses were made in 9 of 16 patients. Biopsy findings resulted in a change in therapy in 11 of 16 patients. Overall, the SLB surgical complication rate was 19% (3/16). SLB did not directly cause the observed operative mortality. The ICU mortality rate was 38% (6/16). Patients who switched therapies after SLB had a trend toward decreased mortality than patients without a change in therapy (27% versus 60%; *P* = 0.299).

**Conclusions:**

In selected critically ill cancer patients with ARDS, SLB had a high diagnostic yield rate and an acceptable surgical complication rate.

## Background

Survival rates for different types of cancer patients have improved in recent decades [1]. As a result of increased survival and more aggressive cancer treatments, patients with cancer have required more frequent admissions to hospital intensive care units (ICU). It has been reported that European ICUs admit patients with cancer approximately 15% of the time. Reasons for admission include postoperative care, sepsis, acute respiratory failure (ARF) and treatment related toxicity [2,3]. Treatment outcomes were comparable between solid cancer patients and the non-cancer population, but the patients with hematological cancers had a higher hospital mortality rate [2]. In pooled studies between 1999 and 2005, cancer patients diagnosed with ARF who required mechanical ventilation demonstrated a high mortality rate of approximately 75% [4].

Cancer patients with ARF without a definite diagnosis have a higher mortality rate than patients with a definite diagnosis [5]. The mortality rate of patients with ARDS has been demonstrated to range from 35% to 60% in the general population [6]. The 28-day mortality rate in neutropenic cancer patients with ARDS was 63% [7]. The presence of ARDS was also independent prognostic factors for hospital mortality in patients with hematological cancers or in solid cancer patients [2]. Furthermore, all cancer patients with ARDS, one of the most severe forms of ARF, had died without a definitive diagnosis (9 out of 9) [5]. Due to individual patient differences, their underlying cancer status, and family concerns, their respective intensivists may hesitate to perform an invasive procedure in these critically ill patients. To improve survival outcomes, aggressive diagnostic techniques that offer a more definitive diagnosis, and subsequently more effective treatment options, may be necessary.

Bronchoalveolar lavage (BAL) is a diagnostic tool used to diagnose infections in the lungs. The diagnostic yield that has led to a change in treatment has been reported to be less than 50% in patients with ARDS [8]. Previous studies have demonstrated surgical lung biopsy (SLB) as a safe and useful diagnostic procedure in ARDS patients [9-11]. In cancer patients with ARF, BAL did not significantly increase diagnostic yield compared with noninvasive tests in a randomized controlled trial [12]. SLB is considered safe and has been recommended for use in patients with hematologic malignancies with diffuse pulmonary infiltrates [13,14]. In those patients with hematological malignancies and unexplained pulmonary infiltrates, improved outcomes following a change in therapy was associated with a diagnosis obtained from SLB [15]. The feasibility of SLB in cancer patients with ARDS remains limited. This retrospective analysis evaluated the utility and safety of SLB in cancer patients with ARDS.

## Methods

### Patients

A retrospective chart review was performed from January 2002 to December 2009 of all patients with ARDS undergoing SLB at the Chang Gung Memorial Hospital, a tertiary care referral center. The study design was approved by the Institutional Review Board, Chang Gung Memorial Hospital. All patients met ARDS criteria as defined by the American-European consensus conference in 1994 [16]. Cancer patients with ARDS who underwent SLB while supported with mechanical ventilation were included. Patients who did not need mechanical ventilator support at the time of SLB were excluded. Decisions to perform SLB were made by senior intensivists in charge of the respective ICUs. The indications of SLB were if ARDS was suspected to result from a noninfectious etiology, without obvious etiology, and with a possible indication for corticosteroid therapy based on clinical presentations with rapid progression, symmetric distribution on chest X-ray, and predominant ground-glass attenuation in high-resolution computed tomography (HRCT) of the chest.

### Data extraction before surgical lung biopsy

Medical records were reviewed and analyzed for the following data: age, sex, underlying diseases, Acute Physiology and Chronic Health Evaluation (APACHE) II scores at admission to the ICU, ALI scores, positive-end expiratory pressure (PEEP), PaO_2_/FiO_2_ ratio at ARDS diagnosis, dates of respiratory failure and biopsy, diagnostic procedures before biopsy(HRCT or BALs), and medications at time of biopsy. The location of BAL sampling was selected on the basis of HRCT findings or on a chest X-ray if HRCT was unavailable. Each specimen was analyzed for cell count following BAL, as well as bacterial examination for *Legionella*, *Mycoplasma pneumoniae*, *Pneumocistis jiroveci*, and *Mycobacteria*, and for fungal, and virological analyses. Specimens were also sent for cytology and iron stain analysis. BAL results were considered positive if at least one microorganism grew to a concentration of more than 10^4^ colony-forming units/ml.

### Surgical lung biopsy

SLBs were performed in an operating room or at the bedside in an ICU by a trained thoracic surgeon. While under general anesthesia, SLB was performed using video-assisted thoracoscopic surgery (VATS) or a 5-cm thoracotomy, dependant on the patient’s tolerance. The lung biopsy site was determined based on results from chest HRCT or chest X-ray. For VATS and thoracotomy, an endoscopic stapler-cutter (Endo-GIA, Tyco Health-care Group or Ethicon EndoSurgery; Johnson& Johnson) was used to secure the pulmonary margins. Any complications following surgery, such as postoperative air leak, pneumothorax, subcutaneous emphysema, bleeding, wound infection, and postoperative respiratory acidosis were recorded. After the lung biopsy, all specimens were swabbed for aerobic and anaerobic bacteria, fungal, and mycobacterial cultures. Each tissue specimen was cultured and examined by a pulmonary pathologist.

### Data collection after surgical lung biopsy

Results regarding complications of SLB, pathological diagnosis, and postoperative therapeutic alterations were analyzed. Any change to therapy indicated that SLB results led to the addition of a new therapy or original therapy had been stopped. In addition, outcome parameters, including mechanical ventilation weaning rate, ICU and hospital survival rates, and cause of death, were also evaluated.

### Statistical analysis

All statistical analyses were performed using the SPSS (SPSS for Windows, SPSS Inc., Chicago, IL, USA) statistical package. All values are reported as means ± SD. Differences between subgroups were compared by using the *χ*2 test or Fisher’s exact test when the expected number of events was less than five. The significance level (α) for all statistical tests was set at 0.05, and *P* < 0.05 was considered statistically significant.

## Results

Sixty eight ARDS patients underwent SLB in the past 8 years and 16 cancer patients had underlying solid tumor or hematologic malignancy and were chosen for study inclusion. A total of 16 critically ill cancer patients with ARDS who underwent SLB were enrolled. Baseline characteristics of the patients studied are presented in Table 1. Out of the 16 patients enrolled, 10 (62.5%) had a hematologic malignancy (5 had leukemia and 5 had lymphoma) and 6 patients (37.5%) had solid tumor (5 had lung cancer and 1 had breast cancer). Eight patients (50%) received routine chemotherapy regimens in one month. Two patients presented with neutropenia (neutrophil count <500 cells/mL) and 10 patients (63%) had a chest HRCT to evaluate the severity of disease and to select an appropriate biopsy site before SLB. The decisions not to perform chest CT in 6 patients were based on an assessment of potential risks of transport weighed against the potential benefits by the intensivists.

**Table 1:** Patient characteristics on the day of SLB (n = 16)

**Age, mean±SD**	**55 ± 15**
Male gender, n (%)	12 (75)
APACHE II on ICU admission, mean±SD	21 ± 5
Characteristics of the malignancy, n (%)	
Hematologic malignancy	10 (62.5)
Solid tumor	6 (37.5)
Status of the malignancy at the day of SLB	
Recent diagnosis in 3 months	3 (19)
Stable disease	5 (31)
Progression disease	8 (50)
Chemotherapy in one month, n (%)	8 (50)
HRCT before SLB, n (%)	10 (62.5)
BAL before SLB, n (%)	15 (93.8)
PaO_2_/FiO_2_ ratio (mmHg), mean±SD	143 ± 55
PEEP (cm H_2_O), mean±SD	10.9 ± 2.2
Tidal volume (ml/kg PBW)	7.3 ± 1.2
ALI score, mean±SD	3.1 ± 0.3
Days from ARDS onset to SLB, mean±SD	3.0 ± 1.5
Mechanical ventilator days	10.1 ± 6.6
ICU length of stay	11.3 ± 6.3
Hospital length of stay	31.6 ± 18.6

*SLB*: surgical lung biopsy; *APACHE II*: Acute Physiology and Chronic Health Evaluation II; *HRCT*: high-resolution computed tomography; *BAL*: bronchoalveolar lavage; *PaO2*: arterial partial pressure of oxygen; *FiO2*: fraction of inspired oxygen; *PEEP*: positive-end expiratory pressure; *PBW*: predict body weight; *ALI*: acute lung injury.

All 16 patients underwent a flexible bronchoscopy before SLB. One patient did not undergo BAL due to severe hypoxemia during the bronchoscopy. Four patients received BAL prior to ARDS onset and the interval from BAL to SLB was 4.5 ± 0.6 days. The other 11 patients were performed BAL after ARDS onset and the interval from BAL to SLB was 2.4 ± 0.9 days. All microbiological cultures from the BAL in these 15 patients were negative for bacterial, fungal and virological cultures. In one patient who had a diagnosis of *Pneumocistis jiroveci* pneumonia, the cytology of BAL and the histology had the same results. The reason of SLB in this patient was try to find the other possible etiology of ARDS other than *Pneumocistis jiroveci* pneumonia. One patient underwent a transbronchoscopic biopsy before SLB and the biopsy revealed acute and chronic inflammation.

All sixteen patients were intubated and supported with mechanical ventilation after the development of ARDS. For these ARDS patients, pressure control ventilation mode with a low tidal volume protective strategy was applied. Fifteen of 16 patients (93.8%) received empiric antibiotic treatment prior SLB, and 2 patients (12.5%) received steroid therapy before SLB. The days from ARDS onset to SLB were average of 3.0 ± 1.5.

Underlying malignancies, operation methods, pathological findings, treatment alterations, and outcomes are presented in Table 2. Of the 16 patients examined, biopsies were performed from the left lingular lobe, right middle lobe (n = 4; 25%), right lower lobe (n = 3; 19%), and left lower lobe (n = 3; 19%). Fourteen patients (88%) underwent SLB in an operating room and 2 patients (12.5%) received bedside SLB in the ICU. For SLB, video-assisted thoracoscopic surgery (VATS) was used in 7 patients (44%), and thoracotomy was performed in 9 patients (56%).

**Table 2:** Underlying malignancies, operation methods, pathological findings, treatment alterations, and outcomes

**Patient**	**Underlying malignancies**	**Operation methods**	**Pathological findings**	**Treatment alterations**	**Outcome**
1	AML, M2 types	thoracotomy	Acute leukemia with lung involvement	chemotherapy	Deceased
2	Diffuse large B cell lymphoma, stage IVB	thoracotomy	Interstitial pneumonitis; *Pneumocystis jiroveci* infection	cotrimoxazole	Survival
3	CML, post BMT	thoracotomy	DAD; *Pneumocystis jiroveci* infection	cotrimoxazole	Deceased
4	NSCLC, stage IIIb	thoracotomy	Intersitial pneumonitis with fibrosis	No	Deceased
5	ALL, post BMT	VATS	DAD, Aspergillosis infection	No	Deceased
6	NSCLC, stage IV	VATS	DAD; metastatic adenocarcinoma	corticosteroids and stop antibiotics	Survival
7	ALL	VATS	Desquamative interstitial pneumonitis	corticosteroids	Survival
8	Breast cancer,	thoracotomy	Interstitial pneumonitis with fibrosis	no	Survival
9	NSCLC, stage IV	thoracotomy	DAD, acute and organizing phase	corticosteroids and stop antibiotics	Survival
10	AML, M4 types	thoracotomy	Bronchopneumonia with organizing change	no	Survival
11	NSCLC, stage IV	VATS	Interstitial pneumonitis with organizing change	corticosteroids	Survival
12	Mantle cell lymphoma stage IVa	VATS	DAD, acute and organizing phase; viral inclusion bodies	corticosteroids and stop antibiotics	Survival
13	NHL, stage IVB	thoracotomy	DAD; *Pneumocystis jiroveci* infection	no	Deceased
14	SCLC, limited stage	VATS	DAD, acute and organizing phase	stop anti-tuberculosis agent	Deceased
15	Mantle cell lymphoma, stage IIIA	thoracotomy	Acute fibrosis and organizing pneumonia	corticosteroids and stop antibiotics	Survival
16	Diffuse large B cell lymphoma, stage IVA	VATS	DAD, acute and organizing phase	corticosteroids and stop antibiotics	Survival

*AML*: acute myeloid leukemia; *CML*: chronic myeloid leukemia; *DAD*: diffuse alveolar damage; *NSCLC*: non-small cell lung cancer; *ALL*: acute lymphocytic leukemia; *BMT*: bone marrow transplantation; *VATS*: video-assisted thoracoscopic surgery; *NHL*: non-Hodgkin lymphoma; *SCLC*: small cell lung cancer.

**Figure 1:**
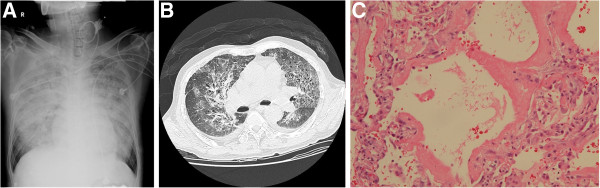
**68-year-old man had mantle cell lymphoma under chemotherapy treatment.** He was admitted to hospital due to fever and respiratory failure. Chest X-ray and HRCT were taken (Figure 1**A** and 1**B**). Because the diagnosis of bilateral lung infiltration remained uncertain after initial evaluation, the patient underwent Video-assisted thoracoscopic lung biopsy. The biopsy result revealed diffuse alveolar damage, hyaline membranes lining the alveolar surfaces (Hematoxylin and Eosin stain, 200X) (Figure 1**C**). The possibility of involvement by the previously diagnosed mantle cell lymphoma was excluded.

All biopsies provided sufficient evidence for a pathological diagnosis with 100% yield. Nine patients (56%) had specific diagnoses: *Pneumocystis jiroveci* pneumonia (n = 3), *Aspergillus flavus* (n = 1), viral pneumonia (n = 1), bacterial pneumonia (n = 1), leukemia with lung involvement (n = 1), metastatic carcinoma (n = 1), and desquamative interstitial pneumonitis (n = 1). Diffuse alveolar damage was identified in 8 patients (50%). An example of pathologic result obtained in the patient with ARDS was shown in Figure 1.

Surgical complications were reported in 3 patients (19%). The first patient had an intraoperative complication with minor bleeding over the staple line and postoperative respiratory acidosis. The second patient had new onset postoperative respiratory acidosis. The third patient had postoperative subcutaneous emphysema. Of the 7 patients receiving VATS, 2 patients had respiratory acidosis with a pH of 7.3 ± 0.2 and PaCO_2_ of 62 ± 32 mmHg within 24 hours after SLB. Overall, the incidence of surgical complications was 11% (1 out of 9) and 29% (2 out of 7) for patients undergoing thoracotomy and VATS, respectively. When SLB was performed in an operating room the surgical complication rate was 21% (3 out of 14). When SLB was performed at the patient’s bedside in the ICU, no surgical complication was reported. No complications following surgery resulted in death.

According to the SLB results, alterations to therapy occurred in 11 patients (69%). The interval between SLB and therapy alteration was 3.4 ± 1.5 days. Corticosteroids were added and antibiotics were discontinued in 5 patients, corticosteroids were added in 2 patients, cotrimoxazole was added in 2 patients, anti-tuberculosis agents were discontinued in 1 patient, and chemotherapy was added in 1 patient. In total, 10 out of 16 patients (63%) were successfully weaned from a mechanical ventilator. The ICU mortality rate was similar to the overall hospital mortality rate (38% [6 of 16]). Patients who required alterative therapy after SLB had a decreased mortality rate compared with patients who did not require alternative therapy (27.3% vs. 60%, *P* = 0.299). The cause of death was severe hypoxemia (n = 3), septic shock with multiple organ failure (n = 2), and intracranial hemorrhage (n = 1).

## Discussion

This small case series study reports that SLB might be considered a useful and safe procedure for cancer patients with ARDS. The rate of treatment alterations made after SLB was increased to 69% (11 out of 16) and patients who required treatment alterations had a decreased mortality rate over patients who did not require a treatment alteration. A definitive diagnosis by using SLB may help intensivists make more informed decisions and offer additional treatment options for patients with cancer and ARDS.

Bronchoalveolar lavage is an invasive diagnostic procedure to identify the cause of ARF in patients with cancer. However, the diagnostic yield of BAL in patients with cancer is not commonly satisfied [4,17]. Recent studies have shown no difference in diagnostic yield between BAL and noninvasive methods when used in patients with malignancies and ARF [12,18]. In the current 15 critically ill cancer patients with ARDS who had received BAL examination, only one patient received a definitive diagnosis by BAL. The possible explanation for the low yield BAL diagnostic rate might be the selection bias because the patients with microbiological diagnosis based on BAL results are unlikely to undergo SLB. The diagnosis for remaining 14 patients who had negative BAL results varied: 5 patients had positive infectious etiology as determined by SLB histology *(Pneumocystis jiroveci* pneumonia [n = 2], aspergilosis [n = 1], bacterial pneumonia [n = 1], and viral pneumonia [n = 1]). Another one *Pneumocystis jiroveci* pneumonia patient was diagnosed by BAL cytology and SLB. Occult infections in those patients determined to be critically ill may be more prevalent than previously thought. The postmortem autopsy findings in critically ill cancer patients showed that the major missed diagnosis was infection [19]. However, if the infection was missed, the precise treatment for the infection would be delayed. In immune-compromised patients with cancer, based on SLB results, it may be necessary to add antibiotics, antifungal, antiviral therapies, or corticosteroids.

ARDS is a clinical-radiographic diagnosis. Some diseases with similar clinical presentations, such as cryptogenic organizing pneumonia, diffuse alveolar hemorrhage, hypersensitivity pneumonitis, and acute eosinophilic pneumonia can mimic and/or cause ARDS and may require other specific treatments [20]. Diffuse alveolar damage (DAD) is the histopathologic finding that corresponds to the clinical entity of ARDS [21]. In a postmortem examination, 50% of 64 patients with ARDS had DAD [22]. In 284 autopsy patients with risk factors for ARDS, the sensitivity and specificity of the criteria of ARDS was only 76% (CI, 67% to 83%) and 75% (CI, 68% to 81%), respectively [23]. The current results demonstrate that 50% (8 out of 16) of cancer patients with ARDS diagnosed clinically actually had DAD. It has been reported that a diagnosis of DAD is found in approximately 40-56% of ARDS patients [10,11].

In a study examining critically ill patients with cancer and ARF, the lack of a definitive diagnosis is predictive of poor survival [5]. A retrospective study that examined 63 patients with hematologic malignancies demonstrated improved survival when SLB resulted in a specific diagnosis [13]. It has also been reported that in patients with hematologic malignancies and unexplained pulmonary infiltrates, a specific diagnosis based on SLB was associated with a lower mortality rate when compared with a nonspecific diagnoses [15]. It is crucial to make a definitive diagnosis as early as possible using aggressive diagnostic techniques for patients with cancer and ARDS. The results of this study showed that 56% (9 out of 16) critically ill cancer patients with ARDS had a specific diagnosis after SLB. This result suggests that SLB is a useful diagnostic tool in critically ill patients with cancer and ARDS.

For hematologic malignancies in non-ARDS patients, some studies have suggested SLB is useful for diagnosis and modifications to treatment, but that its effect on survival remains unclear [24,25]. In the current study, the patients with cancer and ARDS demonstrated a rate of therapy alteration after SLB of 69% (11 out of 16); similar results have been previously reported in ARDS patients (range: 60% to 78%) [10,11,26]. For ARDS patients receiving SLB, previous retrospective studies could not determine the benefit of SLB in terms of survival [9-11]. However, a prospective study in 100 patients with unresolved ARDS showed that survival was significantly improved with contributory SLB than with noncontributory SLB (67% versus 14%, *P* < 0.001) [26]. In the current study, the overall survival rate was 62% and patients who required a change to their treatment after SLB demonstrated increased survival compared with those patients without a change to their treatment (72.7% versus 40%, *P* = 0.299). The most common treatment alteration in this study was antimicrobial therapy adjustment, especially in discontinuing antibiotics (6 out of 11). Discontinued antibiotics could avoid side effects of antibiotics, drug and drug interaction and bacterial resistant strain developed.

The reported rate of complications associated with SLB in ARDS patients have ranged from 17% to 39% [9-11]. The reported rate of complication associated with postoperative SLB was 19% (3 out of 16) in the current study. Among these three patients with postoperative complications, two patients presented with respiratory acidosis after SLB. Postoperative respiratory acidosis is a risk factor for postoperative hypotension. The causes of respiratory acidosis in these 2 patients may have been due to hypoventilation in one lung during the VATS surgical procedure. Another possible explanation for respiratory acidosis was a change to the ventilator setting after the operation. To prevent postoperative air leakage from developing, we routinely decreased the airway pressure by reducing PEEP 2 cmH_2_O from the baseline post-surgery. For ARDS patients with high PEEP, the reduction of PEEP levels may cause the collapse of alveoli and hypoventilation. No death was attributed to SLB in the current study. The risk for complications resulting from SLB in patients with cancer was therefore acceptable, even for the most critically ill patients with ARDS.

Limitations of the current study should be addressed. First, this is a single medical center experience, not a generality. Second, the current study was retrospective with relatively small sample size, the patients who received SLB were not randomized and there was no control group. It is difficult to determine whether SLB is predictive of a survival benefit in cancer patients with ARDS. Nevertheless, understanding specific etiology would allow the introduction of specific therapy if such a therapy is available. Second, the results of this study cannot be generally applied to all ARDS patients. The decision to perform SLB was selective and patients who were referred for SLB were unlikely to be representative of the larger ARDS population. This selection bias caused by patients and intensivists would likely increase the possibility of an alternative treatment. Finally, some specific diagnosis such as diffuse alveolar hemorrhage and viral pneumonitis may be underdiagnosed or delayed because a definitive diagnosis depends on the availability of laboratory facilities. A standardized comprehensive diagnostic examination of BAL before SLB should be established in the future.

## Conclusion

This retrospective study concluded that SLB had a high diagnostic yield rate and an acceptable complication rate for selected cancer patients with ARDS. The rate of treatment alteration after SLB was high (up to 69%) and patients who required treatment alterations had reduced mortality rate than those patients who did not require a treatment alteration. The surgical complication rate of SLB was low (19%) and considered acceptable. It can’t conclude that SLB itself can reduce the mortality. Furthermore, a prospective, randomized controlled trial to investigate the benefit of SLB on survival outcome in cancer patients with ARDS is needed. However, such a study is unlikely to be performed because SLB in cancer patients with ARDS is very low frequency (16 patients in 8 years).

## Abbreviations

ARF: Acute respiratory failure; APACHE: Acute physiology and chronic health evaluation; BAL: Bronchoalveolar lavage; DAD: Diffuse alveolar damage; HRCT: High-resolution computed tomography; ICU: intensive care units; PEEP: Positive-end expiratory pressure; SLB: Surgical lung biopsy; VATS: Video-assisted thoracoscopic surgery.

## Competing interests

The authors declare that they have no competing interests.

## Authors’ contributions

CHC and KCK conceived and designed the study and wrote the article. CCH and NHC recruited patients. The study was performed by HCH, CYH, LFL, CYW, CWW, JYF, and CTY. The data was coordinated and analyzed by YHT. All authors read and approved the final manuscript.
